# 

**Published:** 1918-07-27

**Authors:** 


Mrs. Jessie Smith, of Glasgow, bequeathed, to be paid after the death of her sister, £10,000 to Glasgow Royal Infirmary, to endow a ward in affectionate remembrance of testatrix's late husband, Mr. Finlay Smith; £5,000 to the Western Infirmary, Glasgow; £5,000 to Victoria Infirmary, Glasgow; £2,000 to the Royal Samaritan Hospital for Women, Glasgow; £500 to the Glasgow Magdalene Hospital, £2,000 to the Ayr County Hospital, and varying sums to other charities. The disposal of the remainder of the estate is left to the discretion of the trustees to aid by means of money grants charitable institutions with headquarters in Lanarkshire and Ayrshire which derive no support from rates and taxes.

### HOLIDAYS FOR "RAID" CHILDREN.

The Local Government Board is anxious to send away into the country for a short period a number of children from the East End of London who are suffering from the effect of air-raids, and the Metropolitan Asylums Board has at the request of the Local Government Board arranged to accommodate children at the Children's Home, Hanwell. The movement for sending such children originated with a committee in Poplar.

### Matrons' and Sisters' Appointments.

GENERAL HOSPITAL, NOTTINGHAM. — Miss Winifred McMullen and Miss J. Morgan have been appointed night and out-patient sister respectively. Miss McMullen was trained at the Hertford County Hospital, later being sister there, and has also been temporary night sister at Charing Cross Hospital and ward sister at the Children's Hospital, Bradford. Miss Morgan was trained at the General Hospital, Wolverhampton, and has been night sister at the General Hospital, Weston-super-Mare, and charge sister at the Hospital, Rugby.

PADDOCK MILITARY HOSPITAL, OSWALDTWISTLE.—Miss Mary A. Duckworth has been appointed sister. She was trained at the Selly Oak Infirmary, Birmingham, and has been sister at the Beaconwood Convalescent Hospital, Rendall, Birmingham, and at the Nevill Park Hospital, Tunbridge Wells.

ROYAL INFIRMARY, LEICESTER.—Miss Ella H. Cozens has been appointed second massage sister. She was trained at the Bristol Royal Infirmary, and holds the certificate of the Incorporated Society of Trained Masseuses.

ST. JOHN HOSPITAL, THE GRANGE, SOUTHPORT.—Mrs. Ella Williams has been appointed night superintendent. She was trained at the Victoria Hospital, Blackpool, has been ward sister at the Auxiliary Military Hospital, Levenshulme, Manchester, and theatre and ward sister at Croesnewydd Auxiliary Military Hospital, Wrexham.

SUTTON RED CROSS HOSPITAL.—Miss Jessie Alexander has been appointed matron. She was trained at Preston Royal Infirmary and Glasgow Royal Infirmary, at which latter institution she has been assistant matron. She has also been lady superintendent and matron of the Royal Alexandra Infirmary, Paisley.

WOODLANDS V.A.D. HOSPITAL, SOUTHPORT.—Miss F. V. Buchanan has been appointed night superintendent. She was trained at Charing Cross Hospital, where she afterwards held the post of sister. She has since been sister of the military ward of the East Suffolk Hospital, Ipswich.

**NOTE.—Particulars of Appointments for insertion in this column will be welcomed.**

